# Effect of a combination of carnosine and aspirin eye drops on streptozotocin - induced diabetic cataract in rats

**Published:** 2009-10-21

**Authors:** Qiong Shi, Hong Yan, Ming-Yong Li, John J. Harding

**Affiliations:** 1 Department of Ophthalmology, Tangdu Hospital, Fourth Military Medical University, Xi’an, China; 2 Department of Stomatology, No 451 Hospital of PLA, Xi’an, China; 3 Nuffield Laboratory of Ophthalmology, University of Oxford, Oxford, UK

## Abstract

**Purpose:**

To investigate the effect of a combination of carnosine and aspirin eye drops on the progression of diabetic cataract formation induced by streptozotocin (STZ).

**Methods:**

Rats were made diabetic with STZ. Animals in the treated groups received carnosine, aspirin, or a combination of carnosine and aspirin as drops to the eyes. Cataract progression was monitored by slit lamp microscope and classified into four stages. At the end of 8 weeks, the animals were killed and biochemical changes were determined. Blood and urine glucose levels, body weights, food, and intake were also determined.

**Results:**

About 84.4% of the rats responded to the STZ injection. There were statistically significant differences in the stage of cataract of lenses between the untreated and the treated diabetic animals and between the combination and the aspirin group at the 7th and 8th week. There was a significant decrease in the water-soluble protein in the diabetic groups compared with the control group. The three treatments improved the water-soluble protein levels, and the combination treatment had the greatest effect. The levels of thiol were remarkably decreased in the lenses of diabetic rats, except the combination group. The specific activity of glutathione peroxidase (GPx) was increased and the activities of glutathione reductase (GR) and catalase (CAT) were decreased in all the diabetic groups.

**Conclusions:**

The results indicated that carnosine, aspirin, and a combination eye drops are effective against the onset and development of diabetic cataract in rats. Most important, the effect of combination eye drops is better than aspirin only.

## Introduction

Cataract is one of the major causes of blindness worldwide**.** An estimated 16-20 million persons are blind from cataract, although it is curable through cataract surgery with appropriate correction [1-3]. At present, surgical treatment with phacoemulsification and intraocular lens implantation remains the only proven treatment. This, however, is associated with significant cost and is not readily available, especially in developing countries where the prevalence of cataract is the highest [4,5]. Besides possible complications, an artificial lens does not have the overall optical qualities of a normal lens [6]. Visual inconvenience worsens the quality of life; therefore, preventive and nonsurgical approaches to cataract treatment are highly desired due to an increase in the number of patients, in parallel with the growth and aging of the population [5]. It is estimated that a delay in cataract formation of about 10 years would reduce the prevalence of visually disabling cataract by about 45% [7].

Diabetes is one of the most important risk factors for cataract [1,8,9] and cataractogenesis is one of the earliest secondary complications of diabetes mellitus. Since extracellular glucose diffuses into the lens uncontrolled by the hormone insulin, the lens is one of the body parts most affected in diabetes. Possible pathophysiological mechanisms include non-enzymatic glycosylation (glycation) with cross linking of proteins, and oxidative stress [4]. Multiple mechanisms have been implicated in the development of cataract in diabetes. To date, the exact sequence of events that leads to opacification has not been clearly defined. Thus, the relationship of the opacity to the initiating event may be obscure.

Many anti-cataractogenic agents, such as aldose reductase inhibitors, have been described so far, but owing to lack of success in patients, no drug has yet been approved for clinical use [10-13]. Evidence from epidemiological, in vitro, and animal studies has accumulated to support the idea that carnosine and aspirin [14-18] protect against cataract. Carnosine is a naturally occurring, water-soluble dipeptide. It has antioxidant and antiglycating properties, and may be a potential therapeutic agent mainly due to its antioxidant and antiglycating activities [18-23]. Aspirin has been shown to reduce glycation in vitro, and in animal experiments, probably by acetylation of amino groups, and it also may inhibit glycoxidation and AGE-cross-link formation [1]. Aspirin and aspirin-like analgesics have been studied in a variety of model systems including diabetic rats [15-18]. A variety of laboratory and epidemiological evidence supports the benefits of aspirin-like drugs but there has been no clinical trial specifically in patients with cataract [13,24].

Previously, the results from our studies have shown that diabetic cataract in rats could be prevented by carnosine or aspirin eye drops, which readily penetrated into the eye after topical administration without producing plasma drug levels that could lead to systemic side effects [18]. On the basis of the properties of carnosine and aspirin mentioned above, we suspected that a combination of carnosine and aspirin eye drops could be more effective than either of them alone. Therefore, the purpose of this study was to investigate whether using eye-drops containing carnosine and aspirin together might inhibit the progression of diabetic lens opacities better than eye drops containing carnosine or aspirin alone.

## Methods

### Materials

streptozotocin (STZ) and carnosine were obtained from Sigma Chemical Company (Beijing, China). Sprague-Dawley rats were provided by Animal Laboratories of the Fourth Military Medical University (Xi’an, China). Protein and enzyme quantification kits were obtained from Jiancheng Biology Company (Nanjing, China). Glucotrend 2 was from Roche Diagnositic Limited Company (Xi’an, China). Tes-Tape was from Zhujiang Biochemistry Reagents (Guangzhou, China). All other chemicals and solvents were of analytical grade and were obtained from local companies.

### Animals

One hundred-two male Sprague-Dawley rats, one-month old, initially weighing 135-180 g were used in the study. All the animals were fed standard diet ad libitum and randomly assigned to five groups. The animals were housed in five individual cages in a room. All the animals had free access to drinking water. Group A rats (normal control, n=12) received an injection of 0.02 mol/l citrate buffer (pH 4.5, 65 mg/kg body weight, intraperitoneally) as a vehicle, however, the experimental groups (Group B–E) received an intraperitoneal injection of 1% STZ in citrate buffer at a dose of 65 mg/kg body weight. After 96 h, random blood glucose levels were monitored. The animals having blood glucose levels <14mmol/l were excluded from the experiment and the rest were distributed into the following groups: B (diabetic rats untreated, n=21), C (diabetic rats treated with carnosine eye drops 1% only, n=17), D (diabetic rats treated with aspirin eye drops 0.05% only, n=21), and E (diabetic rats treated alternately with carnosine 1% and aspirin 0.05% eye drops, n=17).

### Experimental design

Carnosine eye drops (1%, pH 7.4) were prepared in 25 mmol/l sodium phosphate buffer (pH 7.4, containing 2.527 g of sodium dihydrogen phosphate dihydrate, 1.36 g of disodium phosphate dodecahydrate, and 800 ml double distilled water). Animals from group C and D were treated respectively by instillation of 1% carnosine eye drops, or 0.05% aspirin eye drops, one drop, twice a day for 8 weeks. Rats from group E received combined treatment by instillation of 1% carnosine eye drops and 0.05% aspirin eye drops twice a day alternately, respectively for 8 weeks. However, the animals from group B only received the instillation of the vehicle solution, one drop, twice a day.

Pupils were dilated before slit lamp examination of lenses. Each rat was checked for cataract every week after streptozotocin injection. Initiation and progression of lenticular opacity was graded, double-blind, according to the Oxford system [15,25]: grade 0, clear; grade 1, wide sutures; grade 2, nuclear with opacities radiating from sutures; and grade 3, dense nuclear without clefts. The stage of cataract was scored according to the classification described above. Blood was collected from the caudal end of rats for glucose estimation with Glucotrend 2 every two weeks. Furthermore, the food intake, water intake, urine weight (daily) and body weights, and urine glucose (weekly) were monitored.

In the 8th week, the animals were killed following cervical dislocation under ether anesthesia, the eyes were enucleated, and the lenses were dissected separately and kept at -70 °C until further analysis. All the biochemical parameters were analyzed in the soluble fraction of the lens homogenate.

### Biochemical estimations:

#### Protein determination

Protein concentration was determined by the Coomassie brilliant blue method using a protein assay kit from Jiancheng Company (Nanjing, China) [26,27]. Two lenses in each rat were ground in 0.9% neutral normal saline (1:9) and homogenized by hand in the ice-water mixture to make the 10% homogenate, and then centrifuged (11,500× g) in Eppendorf tubes. Clear supernatant was used for protein determination, which was carried out according to the method described with the kit's-Coomassie brilliant blue method.

#### Thiol (from glutathione [GSH] and protein) determination

Thiol was measured using the dithio-bis-nitrobenzoic acid (DTNB) method at 25 ^°^C and 412 nm [28-30]. The clear supernatant liquid used for protein determination was taken by suction from the centrifuged Eppendorf tube, and the thiol content was determined according to the description of kit. Thiol reacts with dithio-dinitrobenzoic acid to give a yellow compound, which has a high absorption of light at 412 nm which was measured. Through this colorimetric method the content of thiol in each lens was determined.

#### Assay of GR activity

Glutathione reductase (GR) was determined using an assay kit from Jiancheng Company (Nanjing, China). GR activity was measured according to the procedure in which oxidized glutathione (GSSG) was reduced to GSH catalyzed by GR with NADPH as cofactor. The decrease in the optical density at 340nm was recorded at 25 ^°^C for 2 min. The units of enzymatic activity were calculated using an extinction coefficient of 6.22 mM•cm^-1^ for NADPH. One unit was equivalent to the oxidation of 1 mmol of NADPH per min [31].

#### Assay of CAT activity

Catalase (CAT) was determined using an assay kit from Jiancheng Company (Nanjing, China). CAT activity in lens was assayed with hydrogen peroxide as substrate using a method based on the direct measurement of H_2_O_2_ decomposition. The final volume of each enzyme assay was 3 ml substrate and 20 μl supernatant of lens homogenates. Assay was performed at 25 ^°^C and 24 nm. Enzyme activity was expressed as units per g of protein and one unit of CAT activity represented 1 mmol H_2_O_2_ decomposed per min [32].

#### Assay of GPx activity

Glutathione peroxidase (GPx) catalyzes the reduction of hydroperoxides, including hydrogen peroxides, by reduced glutathione and functions to protect the cell from oxidative damage. With the exception of phospholipid-hydroperoxide GPx, a monomer, all of the GPx enzymes are tetramers of four identical subunits [33,34]. Each subunit contains a selenocysteine in the active site which participates directly in the two-electron reduction of the peroxide substrate. The enzyme uses glutathione as the ultimate electron donor to regenerate the reduced form of the selenocysteine [33,34]. The Glutathione Peroxidase Assay Kit measures GPx activity indirectly by a coupled reaction with GR. GSSG, produced upon reduction of an organic hydroperoxide by GPx, is recycled to its reduced state by GR and NADPH. The oxidation of NADPH to NADP^+^ is accompanied by a decrease in absorbance at 340 nm. The rate of decrease in the A340 is directly proportional to the GPx activity in the sample [35].

### Statistical analysis

One-way ANOVA was used for testing statistical significances between groups of data. Individual pair differences were tested by means of Duncan’s multiple-range test. Heterogeneity of variance was tested by the nonparametric Mann-Whitney test. p<0.05 was considered significant.

## Results

Fourteen (14/90) non-responders to STZ (blood glucose measured <14 mmol/l) at 96 h after receiving an intraperitoneal injection were found; two rats (from Group D and E, respectively) whose blood glucose level had fallen below 14 mmol/l by the 3rd week and the animals that died before the end of the experiment were all excluded, leaving 51 rats in the study.

**Figure 1:**
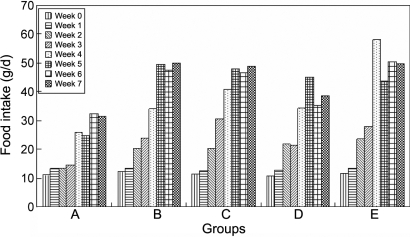
The effect of carnosine, aspirin, and a combination of both eye drops on food intake of each rat per day in various groups, as a function of time, indicating that the food intake in all the diabetic groups increased more than that in the normal group although the normal group's food intake also increased due to its growth. Panel **A** was the normal control group, panel **B** was the diabetic rats untreated group, panel **C** was the diabetic rats treated with carnosine eye drops (1% only) group, panel **D** was the diabetic rats treated with aspirin eye drops (0.05% only) group, and panel **E** was the diabetic rats treated alternately with carnosine (1%) and aspirin (0.05%) eye drops group.

**Figure 2:**
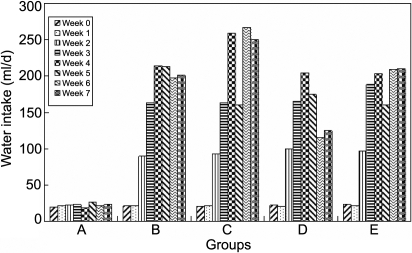
The effect of carnosine, aspirin, and a combination of both eye drops on water intake of each rat per day in various groups, as a function of time, indicating that the water intake in all the diabetic groups increased more than that in the normal group although the normal group's water intake also increased due to its growth. Panel **A** was the normal control group, panel **B** was the diabetic rats untreated group, panel **C** was the diabetic rats treated with carnosine eye drops (1% only) group, panel **D** was the diabetic rats treated with aspirin eye drops (0.05% only) group, and panel **E** was the diabetic rats treated alternately with carnosine (1%) and aspirin (0.05%) eye drops group.

**Figure 3:**
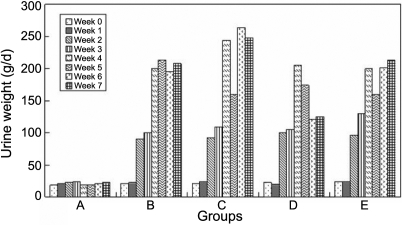
The effect of carnosine, aspirin, and a combination of both eye drops on urine weight of each rat per day in various groups, as a function of time, indicating that the urine glucose in all the diabetic groups increased more than that in the normal group. Panel **A** was the normal control group, panel **B** was the diabetic rats untreated group, panel **C** was the diabetic rats treated with carnosine eye drops (1% only) group, panel **D** was the diabetic rats treated with aspirin eye drops (0.05% only) group, and panel **E** was the diabetic rats treated alternately with carnosine (1%) and aspirin (0.05%) eye drops group.

**Figure 4:**
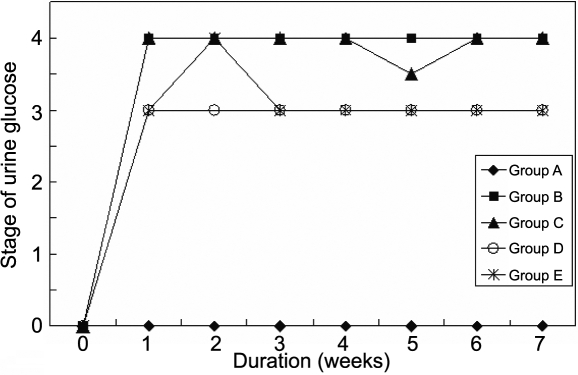
The effect of carnosine, aspirin, and a combination of both eye drops on stage of urine glucose of each rat per day in various groups, as a function of time, suggesting that the progression of cataract appeared slower in all the treated groups. These data indicated that the treatment delayed the the onset of lens opacification. Panel **A** was the normal control group, panel **B** was the diabetic rats untreated group, panel **C** was the diabetic rats treated with carnosine eye drops (1% only) group, panel **D** was the diabetic rats treated with aspirin eye drops (0.05% only) group, and panel **E** was the diabetic rats treated alternately with carnosine (1%) and aspirin (0.05%) eye drops group.

The food (Figure 1) and water (Figure 2) intake in all the diabetic groups increased more than that in the normal group, although the normal group's food and water intake also increased due to its growth, and so did the urine weight (Figure 3) and the urine glucose (Figure 4). The body weight of diabetic rats was much less than that of the control group rats from the first week. The three treatment groups did not improve the body weight of diabetic individuals (Table 1).

**Table 1:** The effect of carnosine, aspirin and a combination of both eye drops on body weight (g/per animal) in STZ-induced rats.

**Group**	**0 week (g)**	**1 week (g)**	**2 week (g)**	**3 week (g)**	**4 week (g)**	**5 week (g)**	**6 week (g)**	**7 week (g)**
A	158.75±9.78 (n=12)	234.17±12.94 (n=12)	280.83±17.17 (n=12)	298.33±22.19 (n=12)	346.25±25.42 (n=12)	390.00±28.20 (n=12)	430.83±46.02 (n=12)	485.42±40.53 (n=12)
B	158.33±13.24 (n=24)	158.57±23.93 (n=21)	146.00±22.63 (n=20)	149.64±28.72 (n=14)	156.15±26.78 (n=11)	165.42±30.49 (n=11)	175.00±29.07 (n=11)	174.09±29.40 (n=11)
C	162.05±11.20 (n=22)	177.06±17.24 (n=17)	172.35±16.50 (n=17)	172.50±20.45 (n=10)	174.00±21.83 (n=10)	175.00±27.89 (n=10)	176.00±30.89 (n=10)	176.50±30.28 (n=10)
D	159.09±10.65 (n=22)	171.90±22.44 (n=21)	143.33±22.55 (n=18)	152.73±12.32 (n=11)	154.09±15.14 (n=11)	156.82±20.65 (n=11)	157.27±24.94 (n=11)	158.64±24.71 (n=11)
E	155.91±12.31 (n=22)	163.82±22.19 (n=17)	169.12±22.31 (n=16)	222.86±13.18 (n=7)	215.00±8.17 (n=7)	205.71±10.18 (n=7)	200.00±17.08 (n=7)	199.29±20.70 (n=7)

The data are mean±SD. “n” was the numbers of the animals in each group every week. There was a statistically significant difference between diabetic rats and normal rats (B~E VS A; p<0.001) from 1 week to 7 weeks. Row A was the normal control group, row B was the diabetic rats untreated group, row C was the diabetic rats treated with carnosine eye drops (1% only) group, row D was the diabetic rats treated with aspirin eye drops (0.05% only) group, and row E was the diabetic rats treated alternately with carnosine (1%) and aspirin (0.05%) eye drops group. The numbers in the 0 week column represent the data of body weight before receiving an intraperitoneal injection.

There was an increase in blood glucose levels in Groups B-E compared with Group A, and the treatment did not reverse the changes in blood glucose levels. The results indicated that treatment with carnosine or aspirin and combination eye drops had no effect on the blood glucose level of STZ-induced diabetic rats (Table 2).

**Table 2:** The changes of blood glucose (mmol/l) after injection of STZ.

**Group**	**0 week (mmol/l)**	**1 week (mmol/l)**	**3 week (mmol/l)**	**5 week (mmol/l)**	**7 week (mmol/l)**
A	7.642±0.8959 (n=12)	7.875±1.1250 (n=12)	7.258±0.8565 (n=12)	5.150±0.7972 (n=12)	6.842±0.8929 (n=12)
B	7.792±0.9427 (n=24)	32.719±1.5468* (n=21)	32.586±2.6726* (n=14)	29.025±5.3268* (n=11)	32.391±3.015* (n=11)
C	7.100±0.7801 (n=22)	30.759±4.1794* (n=17)	32.042±3.5935* (n=10)	24.920±3.1860* (n=10)	30.490±4.535* (n=10)
D	7.368±1.0634 (n=22)	27.040±6.3860* (n=21)	33.118±0.6030* (n=11)	29.345±3.7063* (n=11)	32.027±2.867* (n=11)
E	7.523±1.0752 (n=22)	32.612±1.6959* (n=17)	31.470±3.7589* (n=7)	29.200±3.5473* (n=7)	31.257±3.147* (n=7)

Each value is the mean± SD of results in all the animals in a given group. “n” was the numbers of the animals in each group every week. The asterisk indicates a p <0.001, as compared to the normal group (Group A) with the four diabetic groups (Group B-E). There were no statistically significant differences between the four diabetic groups through the experiment. Row A was the normal control group, row B was the diabetic rats untreated group, row C was the diabetic rats treated with carnosine eye drops (1% only) group, row D was the diabetic rats treated with aspirin eye drops (0.05% only) group, and row E was the diabetic rats treated alternately with carnosine (1%) and aspirin (0.05%) eye drops group. The numbers in the 0 wk column were the data of blood glucose in the different groups before receiving an intraperitoneal injections.

**Figure 5:**
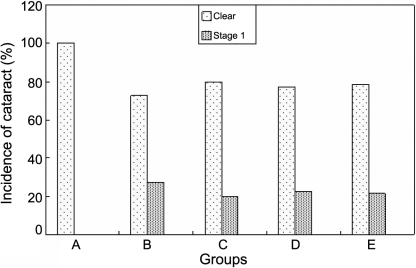
The effect of carnosine, aspirin and a combination of both eye drops on STZ-induced cataract rats at the 5th week. In the calculation of the incidence of lenses in various stages, total lenses in a group were considered to be 100%. Panel **A** was the normal control group, panel **B** was the diabetic rats untreated group, panel **C** was the diabetic rats treated with carnosine eye drops (1% only) group, panel **D** was the diabetic rats treated with aspirin eye drops (0.05% only) group, and panel **E** was the diabetic rats treated alternately with carnosine (1%) and aspirin (0.05%) eye drops group. These data suggested that the combination treatment with carnosine 1% and aspirin 0.05% eye drops delayed the occurrence and progression of STZ-induced diabetic cataract and the effect was better than that with aspirin 0.05% eye drops only.

All the lenses in Group A were clear and normal throughout the study. Eight lenses with an onset of lens opacification were observed at the 4th week in Group B but the onset of lens opacification was not observed until at the 5th week in Group C–E by slit lamp examination (Figure 5).

**Figure 6:**
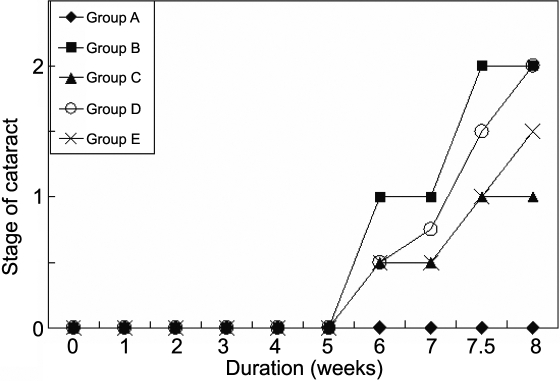
The effect of carnosine, aspirin and a combination of both eye drops on the median stage of cataract, as a function of time. Panel **A** was the normal control group, panel **B** was the diabetic rats untreated group, panel **C** was the diabetic rats treated with carnosine eye drops (1% only) group, panel **D** was the diabetic rats treated with aspirin eye drops (0.05% only) group, and panel **E** was the diabetic rats treated alternately with carnosine (1%) and aspirin (0.05%) eye drops group.

**Figure 7:**
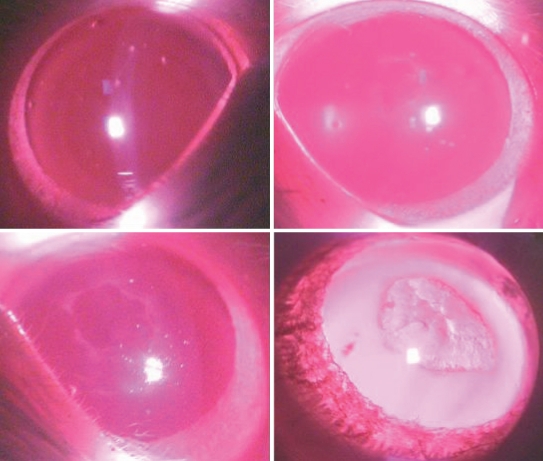
Photographs of the stages of cataract from stage 0 to stage 3 in our study.

**Figure 8:**
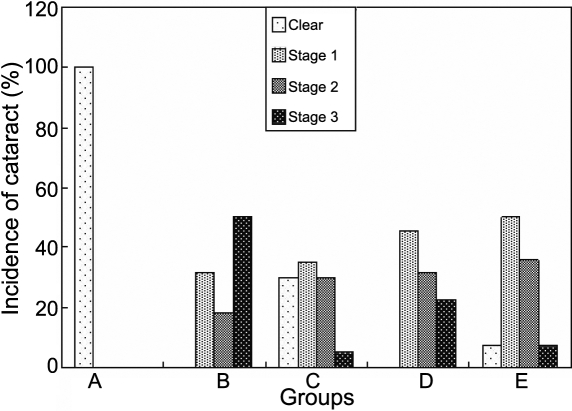
The effect of carnosine, aspirin and a combination of both eye drops on STZ-induced cataract rats at the 8th week. In the calculation of the incidence of lenses in various stages, total lenses in a group were considered to be 100%. Panel **A** was the normal control group, panel **B** was the diabetic rats untreated group, panel **C** was the diabetic rats treated with carnosine eye drops (1% only) group, panel **D** was the diabetic rats treated with aspirin eye drops (0.05% only) group, and panel **E** was the diabetic rats treated alternately with carnosine (1%) and aspirin (0.05%) eye drops group.

The progression of cataract appeared slower in all the treated groups (C, D, and E) compared to the untreated diabetic rats (Figure 6). According to the method of categories reported above, the stage of cataract of lenses among the five groups differed during the duration of the study (p<0.001, Figure 6). Photographs of the stage of cataract from stage 0 to stage 3 in the present study are shown in Figure 7. Moreover, there was a statistically significant difference between the untreated diabetic and the treated diabetic animals (p<0.05), and between Group E and D (p<0.05) at the 7th week and 8th week (Figure 8 and Table 3). Note that at the eighth week the majority of lenses in the untreated diabetic rats reached stage 3 whereas only a minority of rats in the three treated groups reached this stage. These results suggested that the combination treatment with 1% carnosine and 0.05% aspirin eye drops delayed the occurrence and progression of STZ-induced diabetic cataract and the effect was better than that with 0.05% aspirin eye drops only (Table 3). In the 8th week, although the stage of lens opacification in Goup E was higher than that in Group C, no statistically significant difference was found between them (Table 3).

**Table 3:** The effect of carnosine and aspirin drops on STZ-induced lens opacity.

**Group**	**0 week**	**1 week**	**2 week**	**3 week**	**4 week**	**5 week**	**6 week**	**7 week**	**8 week**
A	0 (0~0) (n=12)	0 (0~0) (n=12)	0 (0~0) (n=12)	0 (0~0) (n=12)	0 (0~0) (n=12)	0 (0~0) (n=12)	0 (0~0) (n=12)	0 (0~0) (n=12)	0 (0~0) (n=12)
B	0 (0~0) (n=24)	0 (0~0) (n=21)	0 (0~0) (n=20)	0 (0~0) (n=14)	0 (0~1) (n=11)	0 (0~1) (n=11)	1 (0~2) (n=11)	1 (0~2) (n=11)	2 (1~3) (n=11)
C	0 (0~0) (n=22)	0 (0~0) (n=17)	0 (0~0) (n=17)	0 (0~0) (n=10)	0 (0~0) (n=10)	0 (0~1) (n=10)	0.5 (0~2) (n=10)	0.5 (0~2)* (n=10)	1 (0~3)* (n=10)
D	0 (0~0) (n=22)	0 (0~0) (n=21)	0 (0~0) (n=18)	0 (0~0) (n=11)	0 (0~0) (n=11)	0 (0~1) (n=11)	0.5 (0~2) (n=11)	0.5 (0~2)* (n=11)	2 (1~3) (n=11)
E	0 (0~0) (n=22)	0 (0~0) (n=17)	0 (0~0) (n=16)	0 (0~0) (n=7)	0 (0~0) (n=7)	0 (0~1) (n=7)	0.5 (0~2) (n=7)	0.5 (0~2)*,# (n=7)	1.5 (1~3)*,# (n=7)

Values are expressed as medians (range of grades), “n” was the numbers of the animals in each group every week. Median representation: 0, clear; 1, clear nuclear with wide sutures; 2, slight dense nuclear with opacities radiating from sutures; 3, dense nuclear without clefts; 4, dense nuclear with clefts; 5, nuclear cataract with clefts; 6, nuclear cataract with dense radial opacities; 7, nuclear cataract with whole lens opacities. Statistical analyses were performed by using Wilcoxon Rank Sum Test. The asterisk indicates significantly different from Group B; p<0.05 and the sharp (hash mark) indicates significantly different from Group D; p<0.05. Row A was the normal control group, row B was the diabetic rats untreated group, row C was the diabetic rats treated with carnosine eye drops (1% only) group, row D was the diabetic rats treated with aspirin eye drops (0.05% only) group, and row E was the diabetic rats treated alternately with carnosine (1%) and aspirin (0.05%) eye drops group. Stage 0.5 indicates that about half of the lenses were at stage 0 while the remainder had progressed to stage 1. Stage 1.5 indicates that about half of the lenses were at stage 1 while the remainder had progressed to stage 2. The numbers in 0 wk column represent the data in the lenses before receiving an intraperitoneal injection.

In our previous studies we evaluated the safety of eye drops by observing the extent of corneal staining [18]. In this combination study, no further corneal staining or side effects were noticed.

Protein denaturation has been considered to be the ultimate change that results in lens opacification. Therefore, we analyzed the water-soluble protein content in all the groups. There was a significant decrease in water-soluble protein in group B-E compared with the control group (Table 4). The three treatments appeared to improve soluble protein levels, and the Group E treatment appeared to provide greater improvement than the others. However these differences were not statistically significant.

**Table 4:** The effect of carnosine, aspirin and a combination of both eye drops on soluble protein, thiol content, and the activities of GPx, CAT, and GR in the lens.

**Group**	**n**	**Protein (g/l)**	**Thiol (mg/gl protein)**	**GPx (U/g protein)**	**CAT (U/g protein)**	**GR (U/g protein)**
A	12	10.46±0.69	118.13±24.36	17.28±5.19	75.61±24.75	3.81±2.15
B	11	8.19±1.08*	90.08±18.73*	46.14±4.77*	42.12±10.16	2.75±1.24
C	10	8.53±0.91*	97.95±22.11*	31.31±5.06*#	68.69±21.97	3.66±0.84
D	11	8.63±0.50*	92.38±7.18*	34.38±4.64*#	63.23±25.60	3.23±1.65
E	7	8.90±0.84*	100.89±13.56	26.21±6.99*#**	43.23±9.82	3.63±1.61

The data are mean±SD. “n” was the numbers of the animals in each group. The asterisk indicates significantly different from Group A; p<0.05. The sharp (hash mark) indicates significantly different from Group B; p<0.05. The double asterisk indicates significantly different from Group D; p=0.001. Row A was the normal control group, row B was the diabetic rats untreated group, row C was the diabetic rats treated with carnosine eye drops (1% only) group, row D was the diabetic rats treated with aspirin eye drops (0.05% only) group, and row E was the diabetic rats treated alternately with carnosine (1%) and aspirin (0.05%) eye drops group.

Thiol levels of were decreased in the groups of diabetic lenses. This result indicated that there was increased oxidative stress in diabetic cataractous lenses. Thiol levels were higher in the treated groups although these differences were not statistically significant. Group E thiol levels were improved compared with the other diabetic groups and not statistically significant from the normal control group (Table 4). Although the levels of thiol in Group E had not returned to normal, they improved significantly with treatment when compared with levels in the untreated diabetic animals.

The specific activity of GPx increased significantly in all the diabetic groups (Table 4), but was significantly decreased in treated groups compared to the untreated diabetics. The combined treatment restored GPx levels better than aspirin alone.

There appeared to be decreased activities of GR and CAT in diabetes (Table 4), but there was no statistically significant difference in activity of GR and CAT between the groups.

## Discussion

Cataract is the major cause of blindness and visual impairment worldwide, and multiple factors are involved in the formation of cataract [4,36,37]. Diabetes mellitus is one of the major risk factors for cataract development [9]. Although, there have been major advances in the control of hyperglycemia through dietary changes, hypoglycemic agents, insulin, and islet transplantation, the long-term complications of diabetes, such as cataract, remain serious problems. Various mechanisms have been proposed to explain the pathophysiology of diabetic complications. These mainly include oxidative stress, increased polyol pathway or osmotic stress, and increased formation of advanced glycation end products [36].

Although there is cross talk between these pathways, results in several studies suggest that glycation and oxidative stress contribute the major determinants in diabetic complications [36,38,39]. Many drugs have been used for anticataract research in animals [1,4,12,13,37,40], and some have proven effective in the prevention of lens opacification [4,14-16,18,25].

Carnosine was the first and the most simple example of an active peptide used to prevent diabetic complications. It has recently attracted much attention as a naturally occurring antioxidant and transition-metal ion sequestering agent. The anti-ageing effect of carnosine had been demonstrated in many studies in vivo and in vitro [19-23,41]. In our previous study, there was some evidence verifying the effect of carnosine on preventing cataract development [18]. Through its distinctive combination of antioxidant and anti-glycating properties, carnosine is able to attenuate cellular oxidative stress and can inhibit the intracellular formation of reactive oxygen species and reactive nitrogen species [42]. By controlling glycation, oxidative stress, and by chelating metal ions, carnosine is able to reduce harmful sequelae such as DNA damage [42]. Therefore, the effect of carnosine on delaying cataract formation may be through preventing glycation and oxidation, thus protecting protein and DNA against cross-linking and other damage.

In our present study, the activity of GPx was raised in diabetes and this was partly normalized by carnosine. If the rise can be considered as a reaction to oxidative stress then presumably carnosine decreased this stress. CAT and GR appeared to be decreased in the untreated diabetic rats, but less so in the treated diabetic rats, however there were no statistically significant differences.

Carnosine eye drops, on mice, rats, rabbits, and dogs were well tolerated at both total and local levels [43]. In animals the eye drops did not affect the diameter of the pupil, nor did they increase intraocular pressure. In our study, eye irritation was not noticed [18]. Thus, this dose of carnosine used as eye drops is safe.

Forty years ago, Cotlier [44] reported that aspirin (acetylsalicylic acid) could protect patients with diabetes mellitus against cataract. Experimental studies then showed that aspirin protected lens proteins against a variety of chemicals relevant to cataract formation [17,45-48]. In those experiments, aspirin delayed experimental cataract in laboratory animals by decreasing glycation of lens proteins, and a fall in glutathione levels. The loss of enzyme activity, such as GR, induced by diabetes was also alleviated by aspirin [15,48,49]. The decrease in glutathione and the related enzymes, and the increase in glycation were related to the progression of lens opacification. In the present study, levels of thiol were decreased in the lenses of rats receiving the aspirin eye drops, and the results supported the viewpoint reported above. It has been shown that aspirin reduced glycation in experimental animals by acetylation of amino groups.

Therefore, we considered whether the combination of carnosine and aspirin eye drop treatment might increase the benefits of preventing cataract development. The present results showed that the combination treatment delayed the progression of diabetic cataract in rats. The decrease in soluble protein content in the diabetic group lenses compared with lenses in the normal group could be partly due to insolubilization of proteins and perhaps some leakage. The combination treatment not only mitigated the decrease in water-solution proteins but also mitigated the decrease in thiol levels, and the increase in activity of GPx, some more than other treatments. Furthermore, the effect of combination treatment was greater than carnosine or aspirin eye drops alone in preventing the thiol and related enzyme levels, involving the formation of diabetic cataract. It is possible that the delay of STZ-induced cataract by the combination treatment is partly due to a synergism of carnosine and aspirin.

Carnosine preferentially reacts with sugars to competitively inhibit the glycation of lysine in crystallins. The presence of carnosine inhibits the generation of crosslinked protein, the disappearance of crystallins, and the production of low-molecular-weight species. It is conceivable, therefore, that carnosine can react with polypeptide carbonyl groups, irrespective of their mode of generation, to form protein-carbonyl-carnosine adducts in vivo via a process that might be termed protein “carnosinylation” [50]. Aspirin decreased glycation in vitro [46,51] and in vivo in rats [48,52] and also partly maintained GSH levels in the lenses of streptozotocin-induced diabetic rats [18,52]. This protective action appears to be brought about by acetylation of vulnerable groups of lens proteins [53,54], and then, acetylation of a single lysine in human crystallin was identified [55]. Our present study suggested that carnosine, competitively inhibiting the glycation of lysine, and aspirin, acetylating lysine in crystallins, are synergistic.

Furthermore, if carnosine and aspirin eye drops influenced different changes in STZ-induced hyperglycemia, then a combination of two anti-cataractous drugs could be more effective than either of them alone. Hence, we designed the study to investigate the role of a combination of carnosine and aspirin eye drops in the prevention or delay of STZ-induced diabetic cataract. It is the first time that carnosine eye drops and aspirin eye drops alternately have been used for protecting or delaying diabetic cataract. In the present study, treatment with carnosine and aspirin eye drops, and the combination of both, all delayed the onset and progression of cataract. The above notion was supported by finding that the combination was better than aspirin alone in delaying the progression of lenticular opacity.

Eye drops are better modalities than oral treatment for safety and convenience. The present concentration of eyes drops were based on previous studies [18]. According to our previous experience, the dose of eye drops was chosen to provide equivalent levels of each drug to the lens, and the dose volume was increased along with the increase of body weight. To overcome the limitation of the volume applied by a single dosage to rat eyes, we increased the frequency of administration to sustain the dose volume [18,25]. Therefore, we made carnosine drop solutions to 1mg/ml (0.1%), applied twice a day and the aspirin drop solution to 0.5 mg/ml (0.05%), twice daily. Note that the combination treatment in fact provided only half the amount of carnosine or aspirin as the separate drug treatment regimes.

Interestingly, the combination was more effective than carnosine alone in maintaining levels of thiol and the related enzyme activities, even though the effect of carnosine eye drops were better on delaying the stage of cataract. And the effects of combination eye drops were better than aspirin eye drops on delaying the progression of cataract. These results thus provide a clue, for the first time, that combination eye drops may have the more significant effect to delay the progression of diabetic cataract.

In conclusion, a combination treatment of carnosine and aspirin eye drops administered alternately twice a day, can inhibit cataract formation and progression in a STZ-diabetic animal model. The combination treatment is better than aspirin treatment alone; and in the alteration of biochemical indicators, better than carnosine treatment. Therefore, the treatment of diabetic cataract, with a combination of carnosine and aspirin eye drops, merits further attention.
